# "Drunk or Dying" Cases

**Published:** 1903-03-07

**Authors:** 


### Editor's Letter-Box.

[Our Correspondents are reminded that prolixity is a great bar to publication, and that brevity of style and conciseness of statement greatly facilitate early insertion.]

### "DRUNK OR DYING" CASES.

The Hon Sydney Holland, Chairman of the London Hospital, writes: Last week "Mr. Holland begged the question," this week "he contradicts himself." But assertion does not make argument, and I wish you had been good enough to show how I had failed to deal with the question or where I had contradicted myself. I have tried my best to answer in no uncertain language.

Now you raise a new argument, that the whole medical profession will agree that I am wrong in making a receiving-room officer responsible for the consequences of not admitting a drunk or dying case in the absence of a provision of emergency beds . . . and you tell us that if we do not provide these emergency wards we shall emphasise the fact that we need a medical superintendent. Now apart from a smothered feeling of irritation at being called callous this is really rather amusing. Has it ever occurred to you that to each of these hospitals is attached an influential staff, and that any representations from the staff are almost at once attended to? I can, of course, only speak for the two hospitals of which I happen to be chairman. Neither the medical council of the London nor the staff of the Poplar Hospital have ever asked for these emergency beds, and I have never heard of the request ever having been refused at any of the other hospitals, and at one of them, I know, it has never been made; and since you suggest that these beds would long since have been provided if these hospitals had had medical superintendents, may I remind you that certainly at two of the hospitals you enumerate as not having these wards there are already medical superintendents, and that they have never insisted on this necessity for emergency beds? Therefore, I am entitled to reply to you, in all courtesy, first, that we probably know our own business as well as anyone criticising from outside; and secondly, that you are assuming too much when you suggest that you are voicing the whole medical profession. I repeat, once more, that an emergency case has a far better chance of being rightly diagnosed and kindly treated if put under observation in a general ward than if sent, as you suggest, "to the basement" or "to some part of the building where a noisy patient will not prove a nuisance," necessarily, therefore, I would add, remote. But (and I have never failed to emphasise this "But") these cases are sometimes, not at all always, a nuisance to other patients, and so we have settled at the London to provide other rooms in good time. When these rooms are built, though other patients may occasionally be less inconvenienced, the so-called "drunk or dying" will, I fear, not be so well looked after or watched as they are now. A new form of "grave scandal" will then be at your disposal. "A man left to choke in a room by himself," "locked in to die," and so on, because it is not to be supposed that in the middle of the night a nurse can for long be taken out of a general ward and sent to sit in these basement or remote wards, or that a house surgeon called up from his bed will be expected to sit for the rest of the night watching a drunken man. An occasional "look in" will be all they will get, and all they do get I expect where these wards are provided.

I am sorry to occupy so much of your space except that I suppose your space is available for matters of hospital interest, and certainly the way you have stigmatised the policy on this matter of the largest and best-managed hospitals in London as a "grave scandal" necessitates in common fairness an opportunity of fully replying.

*** Mr. Sydney Holland's logic is like the sea—it ebbs and flows quite regardless of all human laws and restraints. His first argument is that the London Hospital does make adequate provision for "drunk or dying" cases; he then shows how the present system at his hospital is so inadequate that they have actually accepted a contract for the building of 12 rooms especially for the reception of these cases; and he illustrates the pernicious effects of the present system, when giving the reasons for this new outlay of hospital funds, by remarking on the suffering which is entailed upon the remaining patients when such objectionable cases are admitted, as they are now, into a general ward. The tide of his thoughts then recedes again, and he tells us that when these new rooms are built the so-called "drunk or dying" will not be so well looked after or watched as they are now. An occasional "look in" will be all they will get, and all they do get, Mr. Holland expects, where these wards are provided. He states that an emergency case has a far better chance of being rightly diagnosed if put under observation in a general ward than if sent to some part of the building where a noisy patient will not prove a nuisance. We are left to wonder for ourselves why this should be so. Such things are impossible of course where the administration of a hospital is in efficient and experienced hands. Again, Mr. Sydney Holland considers that when dying cases are sent away it is due to an error of judgment on the part of the receiving officer. He denies that he wishes to shirk any responsibility, but he thinks he would be doing very wrong if he defended a man (the receiving officer) when he had made a mistake. To illustrate this point of mistakes in diagnosis Mr. Holland describes a personal experience. If the most eminent man in the profession can err in these cases under the most favourable circumstances possible for making a correct diagnosis, how does Mr. Holland justify his statement that the relatively young and inexperienced relieving officer is to blame when he makes a mistake in the noise, hurry, and bustle of the busiest hospital receiving-room in London? Is it too much to expect that Mr. Holland will answer this straightforward question in a straightforward manner? We can only repeat what we said before, that diagnosis without some hours of observation is often impossible, and that if the receiving officers are not permitted to keep the patients under observation for a sufficient period of time without admitting them to a general ward, to the detriment of the comfort and welfare of the other patients, the managers of that hospital are absolutely and entirely to blame should a dying patient be in consequence sent away from the hospital.—Ed. The Hospital.

